# Seasonal trends in methylphenidate use: A mirror of misuse or compliance?

**DOI:** 10.4102/sajpsychiatry.v31i0.2391

**Published:** 2025-02-17

**Authors:** Renata Schoeman, Stefan J. Benjamin

**Affiliations:** 1 Department of Leadership, Stellenbosch Business School, Stellenbosch University, Bellville, South Africa

**Keywords:** attention-deficit hyperactivity disorder, methylphenidate, atomoxetine, seasonal trends, temporal variation, diversion, adherence

## Abstract

**Background:**

A steady growth in the use of medication for the treatment of attention-deficit hyperactivity disorder (ADHD) has been evident over the past few decades. While growth attests to increased awareness of ADHD and improved access to diagnosis and treatment, concerns have been raised about poor adherence to treatment and diversion of medication.

**Aim:**

This current study explored the seasonal and/or temporal use of methylphenidate (MPH) in South Africa.

**Setting:**

The study was conducted in South Africa.

**Methods:**

A retrospective database analysis was conducted to examine unit sales of MPH over a 9-year period. The unit sales of MPH were compared to those of atomoxetine for the same period.

**Results:**

Unit sales for MPH peaked in May and October, which coincided with the academic high-pressure periods for school learners and university students. This was most evident for MPH immediate release 10 mg. There was a noticeable decrease in unit sales for MPH during December. Atomoxetine demonstrated much less seasonal variation.

**Conclusion:**

The seasonal and/or temporal use of MPH fluctuates following the academic calendar. These changes are driven by both temporary interruptions of treatment, such as ‘drug holidays’, and the misuse and diversion of MPH for non-medical use. This holds significant implications for interventions to improve ADHD outcomes. It is crucial to balance accessibility to treatment with the prevention of misuse of MPH.

**Contribution:**

Our findings highlight the need to reconsider current policies and regulations regarding the appropriate diagnosis and management of ADHD and the scripting, dispensing and monitoring of MPH.

## Introduction

Studies have established the efficacy and effectiveness of both stimulant and non-stimulant medications in the treatment of attention-deficit hyperactivity disorder (ADHD).^1^ Stimulants, such as methylphenidate (MPH), are considered the gold standard of pharmacological treatment, with meta-analyses indicating moderate to large effect sizes in the reduction of symptoms.^2^ Structural and pharmacological similarity between MPH and drugs, such as cocaine and d-amphetamine, has been demonstrated in the past.^3^ Although the behavioural pharmacological profile of these drugs is very similar, the actual rates of abuse are believed to be much lower for MPH than the latter. Methylphenidate has shown the potential for substance abuse in the laboratory setting, but there is virtually no evidence to date that the drug possesses significant abuse potential in patients who are likely to take MPH for clinical purposes.^4,5^

Methylphenidate is still considered a controlled substance in many regions of the world, with strict measures in place at the manufacturing, scripting, dispensing and usage levels of MPH. In the United States, MPH is a Schedule II substance under the *Controlled Substances Act* enforced by the Drug Enforcement Administration (DEA).^6^ Similarly, the South Africa Health Product Regulatory Authority (SAHPRA) classifies MPH as a Schedule 6 substance that may have ‘a moderate to high potential for abuse or for producing dependence, which necessitates close medical management and supervision and strict control over supply’.^7^

More recently, there has been an increased focus on the non-medical use and diversion of MPH as a significant public health problem, especially in college students. Academic and occupational performance enhancements are commonly cited as motivation for non-medical use in individuals without ADHD – even though research has found little to no evidence for these claims.^8,9^ In South Africa, reports on the temporal use of MPH are sparse, with one study based on a self-report questionnaire indicating an increase in use towards the latter part of the academic year.^10^

In our study, we aimed to provide more insights into the seasonal and/or temporal use of MPH in South Africa, through the analysis of monthly unit sales data by a big pharmacy group over a 9-year period. Understanding the seasonal trends in the use of MPH may assist with policy recommendations, advising on psychoeducational programmes and reducing stigma – ultimately, improving the management of ADHD.

### Literature review

A steady growth in the use of ADHD medications has been evident over the past few decades.^11,12^ This can be attributed to the increased awareness of ADHD, the broadening of the diagnostic criteria of ADHD and the increased focus on ADHD in adults.^13,14^ Despite the overall increase in consumption of ADHD medication, it appears that the use of such medication displays seasonal and temporal variation.

In an audit of monthly total ADHD prescriptions for 7 million individuals conducted in the United States between November 2006 and October 2007, significant seasonality was found with total monthly prescription volumes dropping between 22% and 29% in individuals under 17 years of age during the May to July summer holidays. In contrast, prescribing trends for adults 18 years and older did not exhibit any seasonal variation.^15^ In a database analysis of person-days of ADHD medication consumption for 145 269 individuals in Taiwan (mean age 7.7 years) between January 2000 and December 2011, prescriptions for MPH (both immediate and slow-release formulations) showed seasonal patterns, with a significant drop in July – which coincided with the school holidays.^16^ It is thus evident that adherence is problematic despite improved outcomes likely with consistent treatment of most chronic conditions, including ADHD.

Adherence is defined by the World Health Organization (WHO) as ‘the extent to which a person’s behaviour – taking medication, following a diet and/or executing lifestyle changes – corresponds with agreed recommendations from a health care provider’ (p. 18).^17^ Medication adherence therefore refers to the degree to which the medication taken reflects the prescriber’s intent. Non-adherence may include non-use, overuse, abuse or diversion of medication.

Measurement of saliva drug concentrations as a surrogate biomarker for adherence in 254 children over a period of 14 months was compared with parental verbal reporting in the The Multimodal Treatment of Attention Deficit Hyperactivity Disorder Study (MTA) Cooperative Study.^18^ Only 53.4% of the children were physiologically adherent during the study period, while 24.8% were physiologically non-adherent (i.e. medication was not used consistently). In a 13-year longitudinal study of 166 children, prescription redemption was used as a surrogate for adherence.^19^ The results indicated that only 54% of participants used medication consistently, while 25.4% had at least two periods of treatment interruption. In a subset of the participants (*N* = 89), the median medication coverage was 81% for the first 90 days and decreased to 54% afterwards. In a further study, episodic dosing appeared to exacerbate poor adherence with only 50% of individuals with ADHD still taking their medication 3–5 months into treatment. At 15 months, only 20% of individuals were still adherent (regardless of whether they were prescribed MPH, mixed amphetamine salts or ATX [atomoxetine]).^20^

Studies have also investigated the misuse of ADHD medication. In a clinical sample of 55 individuals with ADHD recruited from speciality clinics and community settings (mean age of 20.8 ± 5 years), 22% overused their prescribed stimulants, with 10% using it to ‘get high’ and a further 31% using it with alcohol or other drugs.^21^ Eleven per cent of participants reported selling their stimulants (i.e. diversion). In another study of university students (*n* = 1738), 55 students reported past-year use of prescribed stimulants for ADHD with 22 (40%) indicating at least one item reflecting misuse with 36% using much, 19% misusing and 19% reporting intentional use with alcohol or other substances.^22^ More recently, in a study of 231 141 Grade 8 to 12 learners at 3284 United States schools, the mean self-reported past-year non-medical use of prescription stimulants was 5.7% (± 6.1%) at school level and 6.0% (± 0.001%) at individual level.^23^ Interestingly, the adjusted odds of an adolescent who attended a school with a high percentage of the learner body who reported stimulant therapy for ADHD had approximately 36% greater odds of non-medical use of prescription stimulants when compared with adolescents who attended a school where no learners used stimulant therapy for ADHD.

Two noteworthy South African studies among students at tertiary institutions spotlighted the issues of off-label prescribing, diversion of prescriptions and illegal trade in MPH. One study (*n* = 252 medical students) found the lifetime use of stimulants for non-medical purposes to be 18%, with 85% of this group using the stimulants within the past year. Only 2% of the participants reported a diagnosis of ADHD. Most students (32%) reported using stimulants to improve their concentration.^10^ A second study surveyed 1761 junior students across degrees.^9^ Of the 585 participants (response rate 33.2%), 66 (11.3%) participants reported past-year use of MPH. Of these, only 18 (27.3%) had a confirmed diagnosis of ADHD. The majority of participants (44; 66.7%) obtained their stimulants through doctors’ prescriptions, 21 (31.8%) from friends without payment and 4 (6.1%) bought it from illegal sources. Of the past-year users, 16 (24.2%) used MPH before consuming alcohol.

Understanding the magnitude and pattern of seasonal and/or temporal use of MPH in South Africa will inform targeted interventions to improve adherence to treatment and reduce the non-medical use and diversion of stimulants.

## Research methods and design

A retrospective database analysis was conducted considering the number of unit sales (dispensed units) of MPH over a 9-year period as captured by a big pharmacy group in South Africa, capturing 23.4% of the market share.^24^ Gathering data from this database offered a nationwide perspective and served as a representative sample of MPH usage by the entire population.

### Study population

Secondary data were collected from the database of the pharmacy chain, capturing all sales and dispensed data for medications available at its 715 in-store pharmacies. This database records information collected by pharmacists and pharmacy staff at the time of medication dispensing. During this operational interaction, details about the dispensed medicine, dispensing date and patient information are recorded. Errors in the information captured at pharmacy level are negligible, as all medicines dispensed must be scanned through the system by barcodes on the packaging, or by manually entered identification codes. These data from various pharmacies are consolidated at a central information centre, allowing software users to access this centralised information and to extract diverse raw datasets for analysis.

### Sampling strategy and data collection

The number of unit sales (dispensed units) for each strength of MPH (10 mg, 18 mg, 20 mg, 27 mg, 30 mg, 36 mg, 40 mg and 54 mg) over a 9-year period measured in months (from January 2014 to December 2022) was extracted. For MPH 10 mg, the number of unit sales (dispensed units) for the immediate-release (IR) and long-acting (LA) formulations was captured separately. The number of unit sales (dispensed units) of ATX for each strength (10 mg, 18 mg, 25 mg, 40 mg, 60 mg and 80 mg), as non-stimulant comparison, was extracted for the same period. Atomoxetine was chosen as a comparator, similar to international studies, as it has no abuse potential and it requires continuous use for therapeutic efficacy.^16,25^ It is therefore suitable as a non-stimulant reference point.

### Data analysis

Systemanalyse Programmentwicklung (SAP) Business Objects BI Platform 4.3 Support Pack 2 Patch 9 Version (14.3.2.4469.9) was used to extract the raw data in Microsoft Excel format. Scatter plots were created to visualise sales and dispensing trends over time. A mixed model ANOVA was employed to compare average monthly sales and to identify specific months with higher or lower sales. To evaluate trends without the influence of additional trading days, the entire data set was normalised to represent a standard trading month of 28 days. Time series Fourier analyses were conducted to explore cyclic trends over the time span of the data to assess and evaluate seasonality within the dataset. The dataset was synchronised with the academic calendar year in South Africa to investigate relationships between the observed trends and periods of active academic preparation further. Finally, protected Fisher least significant difference (LSD) tests were used to determine statistical differences in dispensed units between months.

### Ethical considerations

Ethical clearance to conduct this study was obtained from the Stellenbosch Business School Research Ethics Committee (No. 28167). Permission for data extraction from the database was granted by the pharmacy chain.

## Results

The current study included all unit sales (dispensed units) for each strength of MPH and ATX by the pharmacy chain over 9 years. For the period under investigation, the total number of MPH unit sales exceeded 2 million, with a noticeable percentage (39.77%) of the unit sales movement attributed to the MPH 10 mg stock-keeping unit (SKU). In comparison, the total number of ATX unit sales was 128 710, only 6.24% of the MPH unit sales during the same period (see Table 1).

**TABLE 1:** Rank order of methylphenidate and atomoxetine total unit sales from January 2014 to December 2022.

Ranking	SKU	Unit sales	% of total MPH or ATX unit sales	% of total
1	MPH IR 10 mg	758 357	36.77	34.63
2	MPH 36 mg	371 508	18.02	16.96
3	MPH 54 mg	270 942	13.14	12.37
4	MPH 20 mg	175 068	8.49	7.99
5	MPH 27 mg	163 422	7.92	7.46
6	MPH 18 mg	136 469	6.62	5.44
7	MPH 30 mg	79 245	3.84	3.62
8	MPH LA 10 mg	61 424	2.98	2.80
9	ATX 40 mg	50 081	38.91	2.27
10	MPH 40 mg	45 047	2.19	2.06
11	ATX 60 mg	33 477	26.01	1.53
12	ATX 25 mg	19 876	15.44	0.91
13	ATX 80 mg	13 126	10.19	0.59
14	ATX 18 mg	7 006	5.44	0.32
15	ATX 10 mg	5 144	3.99	0.23

Note: Total MPH = 2 061 482; total ATX = 128 710.

MPH, methylphenidate; ATX, atomoxetine; SKU, stock-keeping unit; IR, immediate release; LA, long-acting.

The average unit sales per month during the 9-year period was 7021.83 (see Table 2). When examining the total unit sales of MPH IR 10 mg, it is noticeable that sales peaked in October at 179.48% of sales during December (the month with the lowest unit sales) and 120.85% of the average sales per month. May was the month with the second highest unit sales at 116.35% of the average sales per month. December showed a considerable drop in sales at 67.33% of the average monthly sales and a mere 57.87% and 55.71% of sales during May and October respectively.

**TABLE 2:** Descriptive statistics for methylphenidate 10 mg immediate release unit sales.

Month	Mean	s.d.	Maximum	Minimum
January	6712	1596	8553	4320
February	6802	1543	8452	4295
March	7264	1611	8793	4498
April	6469	1470	8074	4207
May	8170	1785	10 035	5443
June	6714	1465	8595	4639
July	6842	1466	8742	4639
August	7528	1666	9402	4672
September	7238	1383	8710	5041
October	8486	1654	10 418	5878
November	7309	1399	8983	5028
December	4728	1112	6086	2905

s.d., standard deviation.

**FIGURE 1:**
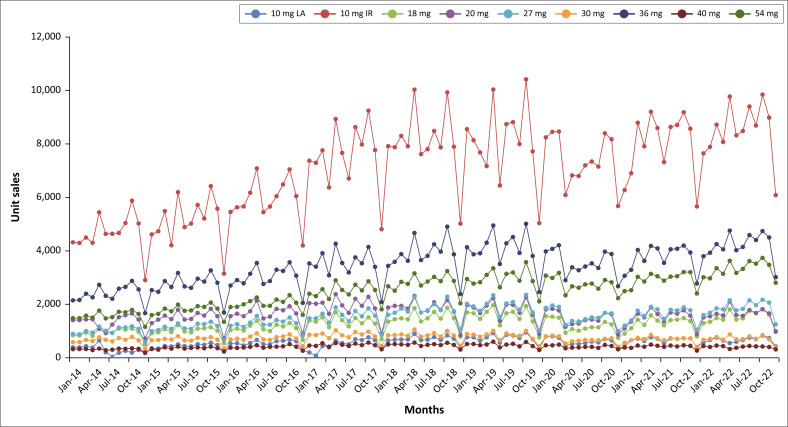
Unit sales for methylphenidate from January 2014 to December 2022.

Figure 1 demonstrates the fluctuations in MPH unit sales for the study period. Although the unit sales of all SKUs fluctuated, the fluctuations were most evident in the MPH IR 10 mg SKU. It appears that these fluctuations coincide with the academic periods for school learners and university students in South Africa, with increased sales prior to the end of terms and semesters (i.e. periods of assessments and examinations) and lower sales during the holidays and early in the academic terms and semesters. The most significant drop in all SKU sales occurred during the December holiday periods.

**FIGURE 2:**
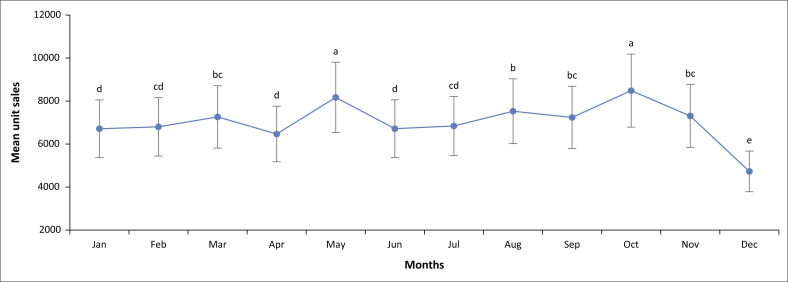
Least squares mean graph for accumulated monthly methylphenidate 10 mg (immediate release) unit sales (*p* < 0.01).

The seasonality in sales of different SKUs becomes more evident when the total unit sales for each month over the entire period are combined. Notable patterns include peak sales in May and October with a significant drop in December each year. Additionally, inflated sales in August and March each year were also observed in the dataset. Although MPH 10 mg LA unit sales also peaked in May and October, the means were similar to March, August, September and November (see Figure 2).

As for the other SKUs (MPH 18 mg, 20 mg, 27 mg, 30 mg, 36 mg, 40 mg and 54 mg), a similar pattern to MPH IR 10 mg (in the least mean square graphs) was observed although May and October were not significantly different from August. All SKUs demonstrated a significant drop in December.

**FIGURE 3:**
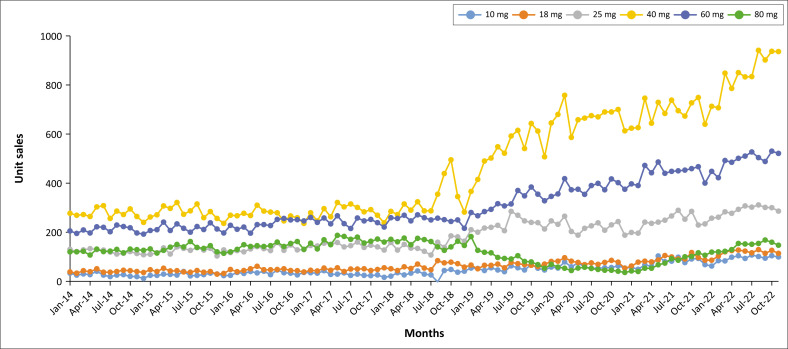
Unit sales for atomoxetine from January 2014 to December 2022.

Figure 3 demonstrated much less fluctuation in the unit sales for ATX than for MPH for the study period. It is interesting to note how the use of ATX gradually increased over the study period – especially for the latter part of 2018.

**FIGURE 4:**
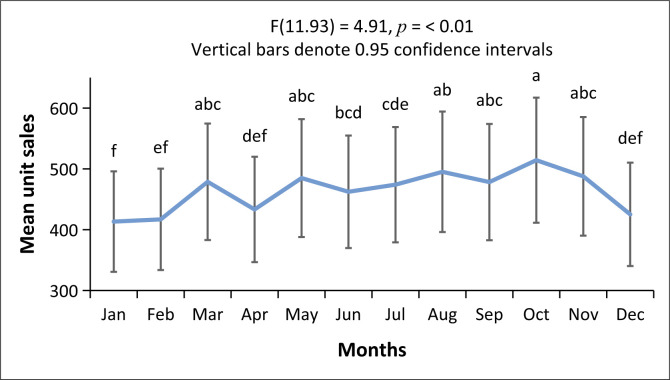
Least squares mean graph for accumulated monthly atomoxetine unit sales (*p* < 0.01).

In contrast with MPH, ATX demonstrated a strong correlation between means for the months of March to November, with significant lower unit sales during December to February (see Figures 3 and 4).

## Discussion

The current study found significant seasonal or temporal changes in the magnitude and pattern of MPH use in South Africa. May and October had the largest number of unit sales (dispensed units), followed by August. These months coincided with the end-of-term and end-of-semester assessment periods in the South African academic year. A significant drop in unit sales was evident during December – coinciding with the summer holiday period. Furthermore, the magnitude of change was predominantly because of the change in the unit sales of MPH IR 10 mg. The sales of ATX, although lower during December to February, were much more stable throughout the years under study.

The seasonality or temporal patterns of use observed in this study are similar to those of international studies. A Turkish study, which investigated prescription of MPH and ATX over a 4-year period, found increased use from March to May (which coincided with academic assessments), with a drop off from June to August (school holidays).^25^ Similarly, in a United States study, spanning a period of 2 years, the drop off in ADHD scripts during the summer holidays was between 22% and 29% – especially in youths below 17 years of age.^15^ In a Taiwanese study, the drop in MPH and ATX use during February and July (the two school holiday periods) was also noteworthy.^16^

The short-term discontinuation of MPH by patients with ADHD is common, with so-called ‘drug holidays’ prevalent in 25% – 70% of families.^26^ This may be the result of parents discontinuing medication for their children, choice in older individuals or recommendation by the treating healthcare professional. Medication is often discontinued for the management of side effects, such as insomnia, anorexia or impaired growth – despite inconsistent evidence for temporary discontinuation.^1^ ‘Drug holidays’ are also thought to allow older children to self-assess their ability to manage without treatment, when they express the wish to ‘be themselves’ or when they want to discontinue medication prematurely.^27^ Many individuals also view medication as necessary only for the academic challenges frequently associated with ADHD.^28^ Another contributing factor in South Africa may be financial reasons – as funding for the treatment of ADHD is limited.^29^

It is interesting to note the gradual increase in unit sales for ATX, especially since the latter half of 2018. This may indicate a gradual increase in the dose prescribed (as children became older, their medication needs in mg/kg increased), as well as the increased confidence of healthcare providers in prescribing ATX – also in the treatment of adults.^30^ The use of ATX showed less seasonal or temporal fluctuation than the use of MPH. This agrees with international studies.^16,25^ The fewer fluctuations in sales may be attributed to the pharmacological effect of ATX requiring at least 6 weeks to take effect and up to 12 weeks for maximum effect.^31^ Even short-term discontinuation of treatment is therefore not advisable. Tolerability of MPH and ATX also differs among individuals, and some may be more willing than others to continue treatment, even during holiday periods.^1,32^

Another explanation for the drop off in unit sales during the summer holidays may be seasonal fluctuations in the severity of symptoms. Research is, however, still contradictory, with some studies reporting an increase in hyperactivity and inattentiveness during spring and summer,^33^ while others found a reduction in inattentive symptoms in summer, which was postulated to be because of seasonal light variability with more circadian rhythm stability during summer.^34^

It would be worthwhile exploring the possible reasons for the peak in unit sales coinciding with the academic high-pressure times. This may be related to better compliance of individuals with ADHD and the clinical use of a so-called ‘top-up’ dose of MPH IR in the afternoon. While this study did not capture data specific to age group or ADHD subtype, hyperactivity and impulsivity tend to decline with age, while inattentive symptoms persist. This may also contribute to the use of MPH during high-pressure academic periods by individuals who are well controlled on ATX for day-to-day functioning. Another driver of the peak in unit sales of MPH IR may be the non-medical use (i.e. off-label use as cognitive enhancer) by individuals without ADHD.

In an informal survey among its readers (1400 participants from 60 countries), *Nature* reported that 20% of respondents said that they had used medication for non-medical reasons – to increase their focus and concentration or to enhance their memory. For those who engaged in this non-medical use of medication, the most popular choice was MPH – used by 62%.^35^ The Global Drug Survey, an annually conducted anonymous web survey on substance use, compared the use of medication for cognitive enhancement in individuals without ADHD over 12 months. Comparing data sets from 2015 (79 640 participants) and 2017 (29 758 participants), the use of prescription stimulants (such as MPH) and illegal stimulants had increased by 180% (4.9% – 13.7%).^36^ The use of MPH as a cognitive enhancer to improve academic or workplace productivity in individuals without ADHD might, however, be based on misperceptions regarding the efficacy of such a strategy. There is a subjective belief that medication, such as MPH, is effective as cognitive enhancers in healthy individuals, but the evidence is ambiguous.

A meta-analysis of studies has indicated that MHP may have dose- and task-dependent effects on some cognitive domains in healthy individuals, such as alertness, attention, verbal memory and executive functioning, as well as reduced fatigue. No consistent benefits were, however, demonstrated for visual memory (of specific importance for students, as this is the domain most important for revision).^37^ Studies have also indicated that MPH can have differential effects based on pre-intervention cognitive capacity, that is, those with lower baseline measurements have more potential for benefit and improvement than individuals who are higher functioning at baseline.^38,39^ In a recent study of the effectiveness of stimulants in enhancing outcomes of complex cognitive tasks, stimulants increased the effort of participants (i.e. decision time and the number of steps taken to find a solution), but productivity (the quality of the solution) decreased simultaneously. There was deterioration in the performance of participants with above average baseline functioning, while those with lower baseline functioning showed some potential for improvement.^40^ It therefore appears that stimulants make one work harder, but not smarter – and may even impair cognitive performance.

Another consideration is the ease of obtaining MPH – despite the (supposedly) strict regulation as a Schedule 6 substance in South Africa. In a quantitative cross-sectional study of students, the most common source for non-medical use of MPH was through diversion – either from friends (30.8% in those with ADHD and 77.3% in individuals without ADHD) or from family members (7.7%). Those without ADHD obtained MPH by buying from pharmacies without a script (14.3%) and through fabricated prescriptions (10.7%).^41^ In a recent study exploring stakeholders’ understanding and perceptions of the scheduling of MPH in South Africa, the current Schedule 6 status of MPH was not considered an effective strategy to prevent misuse and diversion but negatively affected treatment adherence by individuals with ADHD.^42^ Alternative strategies to regulate the use of MPH should be explored.

The current study is the first to explore the seasonal or temporal use of MPH in South Africa. Because of the time span and the market share of the pharmacy chain, it can be deemed representative of the population. The study has clearly identified peaks and troughs in the use of MPH but not for ATX, which coincides with the academic year. These peaks and troughs cannot be attributed to the misuse and diversion of MPH only (as popularly believed) but also to poor adherence to treatment by individuals with ADHD. This highlights the importance of psychoeducation to improve adherence to treatment by individuals with ADHD but also the need to re-evaluate the role of current regulations in terms of the scheduling of MPH.

For future studies, it is recommended to correlate MPH use with confirmation of a diagnosis of ADHD, age-based analysis and regional analysis of MPH use, to narrow down the drivers of non-adherence to treatment further. In this study, unit sales or dispensed units were used as a proxy for actual use. Future studies could use biomarkers to confirm the actual use of MPH.

As reports of individuals with ADHD only using medication ad hoc, as well as the use of MPH by individuals without ADHD could negatively affect the view of ADHD as a ‘real’ disorder, effective strategies to curtail these patterns should be implemented to decrease the stigma associated with ADHD and MPH, improve the management of ADHD and reduce the burden on the healthcare system. It is essential to review regulatory policies associated with prescribing, dispensing and monitoring of MPH to balance accessibility for those with ADHD while limiting the inappropriate off-label use of MPH.

Specific recommendations to improve adherence (both in the appropriate use of MPH by individuals with ADHD and limiting the diversion and misuse of MPH) include psychoeducation regarding the importance of consistent use of psychopharmacological treatment to improve outcomes. Education of the broader learner, student and general population in terms of effective strategies to enhance academic and workplace performance should be prioritised. Examples of such strategies include effective study methods, stress management, time management and self-care and addressing misconceptions regarding the perceived effectiveness of MPH as a cognitive enhancer. Furthermore, regulatory strategies, such as unified digital pharmacy databases and electronic scripting, preferably linked to service providers and electronic health records to confirm dispensing linked to confirmed ADHD diagnosis, may be more effective in curtailing diversion and misuse, without impeding accessibility of treatment and treatment adherence in individuals with ADHD.

## Conclusion

This study is the first to explore the seasonal and/or temporal use of MPH in South Africa. It is evident that the unit sales or dispensed units of MPH fluctuate following the academic calendar in South Africa. It is postulated that these fluctuations are driven by poor adherence as well as non-medical use of MPH.

Our findings highlight and expand on previous international research regarding seasonality in MPH use, as well as the rising concern about diversion and misuse of MPH. This necessitates appropriate psychoeducational programmes in which misconceptions regarding ADHD diagnosis and treatment, and the utility and risks of diversion of MPH can be addressed. Our findings furthermore emphasise the need to reconsider current policies and regulations regarding the appropriate diagnosis and management of ADHD and the scripting, dispensing and monitoring of MPH.

Through this multi-pronged approach and a collaborative approach between stakeholders, stigma regarding ADHD and MPH can be reduced – ultimately improving the outcomes for all individuals with ADHD.
